# 晚期肺炎型肺癌：一项中国单中心临床-放射-病理特征回顾性研究及预后分析

**DOI:** 10.3779/j.issn.1009-3419.2019.06.01

**Published:** 2019-06-20

**Authors:** 永健 留, 霁 李, 世波 王, 闽江 陈, 静 赵, 德利娜 蒋, 巍 钟, 燕 徐, 孟昭 王

**Affiliations:** 1 100730 北京，中国医学科学院，北京协和医学院，北京协和医院呼吸与危重症医学科 Department of Respiratory and Critical Care Medicine, Chinese Academy of Medical Sciences and Peking Union Medical College, Beijing 100730, China; 2 100730 北京，中国医学科学院，北京协和医学院，北京协和医院病理科 Department of Pathology, Peking Union Medical College Hospital, Chinese Academy of Medical Sciences and Peking Union Medical College, Beijing 100730, China; 3 261042 潍坊，潍坊市第二人民医院呼吸与危重症医学科 Department of Respiratory and Critical Care Medicine, Weifang Respiratory Disease Hospital & Weifang No.2 People's Hospital, Weifang 261042, China

**Keywords:** 肺肿瘤, 肺炎型肺癌, 浸润性肺粘液腺癌, Lung neoplasms, Pneumonic-type lung carcinoma, Invasive mucinous adenocarcinoma

## Abstract

**背景与目的:**

肺炎型肺癌是一种临床和影像表现特殊的肺癌。本研究旨在总结此类肺癌的临床、影像及病理学特征，诊断手段，治疗方案及预后情况。

**方法:**

肺炎型肺癌定义为：肺部计算机断层扫描（computed tomography, CT）以磨玻璃或实变影为主要表现，经组织学或细胞学明确诊断的肺癌。收集2013年1月1日-2018年8月30日期间，就诊于北京协和医院呼吸与危重症医学科的晚期肺炎型肺癌病例，回顾性分析这些患者临床资料并进行生存随访。

**结果:**

共纳入46例患者，均为肺腺癌。咳嗽（41/46, 89.1%）、咳痰（35/46, 76.1%）是最常见的临床表现。胸部CT常见表现为磨玻璃影（87.0%）、实变影（84.8%）、以及多发磨玻璃结节（84.8%），多发囊样变和空洞分别为40.0%和13.0%。同侧及对侧肺转移分别见于95.3%和84.8%的病例。从出现症状到明确诊断的中位时间为214天（95%CI: 129-298）。CT引导肺穿刺活检及外科肺活检的确诊率为100%，支气管镜下的支气管肺泡灌洗（BAL）联合经支气管肺活检（TBLB）的确诊率为80.9%（17/21），痰液病理学检查的确诊率为45.0%（9/20）。病理亚型，26例（26/46, 56.5%）为浸润性粘液腺癌，20例患者因缺乏足够的病理标本量而无法进一步区分亚型。38例进行了*EGFR*基因检测，6例（6/38, 15.8%）有突变。33例进行*ALK*基因检测，仅1例（1/33, 3.0%）有*ALK*重排。中位总生存期（overall survival, OS）为522天（95%CI: 424-619）。EGFR野生型或*ALK*融合基因阴性的患者，化疗明显延长中位OS（HR=0.155, *P*=0.002, 2），接受化疗者中位OS 547天（95%CI: 492-602），不接受化疗者中位OS 331天（95%CI: 22-919）。

**结论:**

肺炎型肺癌由于其临床和影像特征与肺部感染相似而经常导致延误诊断。支气管镜下的BAL联合TBLB有相当高的确诊率。浸润性粘液腺癌为肺炎型肺癌的主要病理亚型。肺炎型肺癌*EGFR*突变及ALK重排发生率很低。对于无明确驱动基因的患者，应积极行化疗，延长患者生存期。

## Abstract (en)

**Background and objective:**

Pneumonic-type lung carcinoma is a special type of lung cancer both clinically and radiologically. Here we present our experience on pneumonic-type lung carcinoma in an attempt to investigate the clinical, radiological and pathological features, diagnostic procedures, treatment, and prognosis of this type of tumor.

**Methods:**

Pathologically confirmed lung cancer with a chest CT characterized by ground glass opacity or consolidation was defined as pneumonic-type lung carcinoma. Cases with advanced pneumonic-type lung carcinoma admitted to Peking Union Medical College Hospital (PUMCH) from January 1, 2013 to August 30, 2018 were enrolled. Retrospective analysis of clinical data and survival follow-up of these patients was conducted.

**Results:**

A total of 46 cases were enrolled, all of which were adenocarcinoma. Cough (41/46, 89.1%) and expectoration (35/46, 76.1%) were the most prominent symptoms. The most frequent chest CT findings were ground glass attenuation (87.0%), patchy consolidation (84.8%), and multiple ground-glass nodules (84.8%). Multiple cystic changes (40%) and cavitation (13%) were also quite frequent. Ipsilateral and contralateral intrapulmonary metastasis were noted in 95.3% and 84.8% of cases respectively. The median duration from symptom onset to diagnosis was 214 days (95%CI: 129-298). Both surgical lung biopsy and CT-guided percutaneous lung biopsy had a diagnostic yield of 100%. Transbronchial lung biopsy (TBLB) combined with bronchoalveolar lavage (BAL) had a diagnostic yield of 80.9% (17/21). Sputum cytology had a diagnostic yield of 45% (9/20). Twenty-six cases were invasive mucinous adenocarcinoma (26/46, 56.5%) and the remainder were unable to identify pathological subtypes due to lack of adequate biopsy sample size. *EGFR* mutation was detected in 15.8% (6/38) of patients and *ALK* rearrangement was detected in 3.0% (1/33) of patients. The median overall survival for these patients was 522 d (95%CI: 424-619). In patients without *EGFR* mutation or ALK rearrangement, chemotherapy significantly improved survival (HR=0.155, *P*=0.002, 2). The median overall survival was 547 d (95%CI: 492-602 d) with chemotherapy and 331 d (95%CI: 22-919) without chemotherapy.

**Conclusion:**

Diagnosis of pneumonic-type carcinoma is usually delayed due to clinical and radiological features mimicking pulmonary infection. TBLB combined with BAL has a quite high diagnostic yield. The most frequent histological type is invasive mucinous adenocarcinoma. The incidence of *EGFR* mutation or ALK rearrangement is low in pneumonic-type carcinoma. For patients without cancer driver genes, chemotherapy is recommended to improve overall survival.

肺癌目前是全球死亡率最高的肿瘤^[1]^，其中非小细胞肺癌约占肺癌总数的85%，腺癌在非小细胞肺癌中约占55%^[2]^。在临床实践中，有一种特殊类型的肺癌，肺部影像学表现类似于肺炎，患者以咳嗽、咳痰为主要临床表现，可间断有发热，抗感染后肺内病变无明显吸收，随着病情进展，患者出现明显的咳泡沫样痰，甚至痰量非常的大，严重时可导致呼吸衰竭，最终依靠病理诊断为肺腺癌，临床上常称之为肺炎型肺癌（pneumonic-type lung carcinoma）。这类肺腺癌有独特的临床表现、影像学特征，发病初期由于难以与肺部感染鉴别，往往导致诊断延误，此类肺腺癌病理亚型多为浸润性粘液腺癌，但通常检测不到常见的表皮生长因子受体（epidermal growth factor receptor, *EGFR*）或间变淋巴瘤激酶（anaplastic lymphoma kinase, *ALK*）等肺腺癌驱动基因，治疗方法相对较少。因此，肺炎型肺癌是一种特殊类型的肺癌。

2011年，国际肺癌研究协会/美国胸科协会/欧洲呼吸协会（International Association for the Study of Lung Cancer/American Thoracic Society/European Respiratory Society, IASLC/ATS/ERS）联合颁布了新的肺腺癌病理分型^[3]^，其中一个重要的变化，是将原有的细支气管肺泡癌进行了拆分，因为细支气管肺泡癌包含有多种不同的病理类型（例如粘液性与非粘液性、浸润性与非浸润性），临床表现差异巨大，临床预后截然不同。目前粘液性细支气管肺泡癌的名称已经不再应用，而部分定义为肺浸润性粘液腺癌，属于浸润性腺癌亚型的一种。肺炎型肺癌的影像学表现以磨玻璃影或实变影为特征，其病理多为肺浸润性粘液腺癌，由于肺泡腔内肿瘤细胞及粘液的填充而形成独特的影像表现^[4]^。

本研究对肺炎型肺癌的临床特征进行总结和分析，以期提高对于该病的临床认识，提供最佳诊断措施，并评估治疗方案疗效及预后。

## 1 资料与方法

### 1.1 患者资料

2013年1月1日-2018年8月30日，就诊北京协和医院呼吸与危重症医学科，影像学类似于肺炎，即肺部计算机断层扫描（computed tomography, CT）表现以磨玻璃或实变影为主，并经组织或细胞学明确诊断的肺癌患者，临床分期为不能接受手术或放疗的Ⅲ期患者以及Ⅳ期患者。该研究获得北京协和医院伦理委员会批准。

### 1.2 研究方法

收集患者病例资料，回顾患者临床信息，肺癌相关信息包括：性别、年龄、吸烟状态、肿瘤家族史、临床表现、体格检查、肿瘤标志物、CT影像学表现、正电子发射计算机断层显像（positron emission tomography/CT, PET/CT）表现、病理获得方式、肺癌诊断时间、病理诊断、基因诊断信息、肺癌分期、转移部位、肺癌相关治疗、疗效及转归。

### 1.3 评价标准

肺癌病理诊断基于2011年IASLC/ATS/ERS肺腺癌国际多学科新标准^[3]^。肺癌临床分期依国际抗癌联盟（Union for International Cancer Control, UICC）第七版肺癌TNM分期标准^[5]^。肿瘤疗效评价依据实体瘤疗效评价标准1.1版（Response Evaluation Criteria in Solid Tumours, version 1.1, RECIST 1.1）^[6]^。

### 1.4 统计学方法

计量资料应用均数±标准差（Mean±SD）表示。计数资料采用率表示。以*Kaplan-Meier*法绘制生存曲线，并计算中位无疾病进展生存时间（progression-free survival, PFS）和中位总生存时间（overall survival, OS）。单因素分析应用*Log-rank*检验比较不同组间生存时间的差异。应用SPSS 17.0统计软件进行统计学分析及进行统计学绘图。

## 2 结果

### 2.1 基本临床特征

本研究共纳入46例患者，男:女比例19:27，诊断肺癌的平均年龄为（60.6±10.3）岁，17例（17/46, 37.0%）患者有吸烟史，9例（9/46, 19.6%）患者有肿瘤家族史。

### 2.2 肺部临床表现及体征

咳嗽（41/46, 89.1%）、咳痰（35/46, 76.1%）是最常见的临床表现，咳痰量每日5 mL-500 mL不等。咳痰多的患者，会持续咯大量白色泡沫痰，为该病的特征性临床表现。10例（10/46, 21.7%）患者有呼吸困难，血气分期提示低氧血症，另有3例（3/46, 6.5%）患者有胸痛，12例（12/46, 26.1%）患者病程中曾有间断发热，但没有持续发热，7例（7/46, 15.2%）患者有痰中带血。31例（31/46, 67.4%）患者出现肺部湿罗音，而其他体征不突出。

### 2.3 影像学表现

本组患者肺部CT均为肺炎样表现（图 1），肺部存在磨玻璃影或者肺炎样的实变影，肺部影像学表现列于表 1。最为常见的表现为磨玻璃影（87.0%）及实变影（84.8%）、多发磨玻璃结节（84.8%），可有支气管充气征（80.4%）、枯树枝征（63.0%）。增强CT可见血管造影征（22/34, 64.7%）。大部分患者有胸腔内转移：同侧肺转移（95.3%），对侧肺转移（84.8%），胸膜转移（34.8%）及纵隔淋巴结增大（63.0%）。18例患者完成PET/CT检查，结果提示肺部病变有不同程度SUV值增高（1.9-14.1）。

**1:**
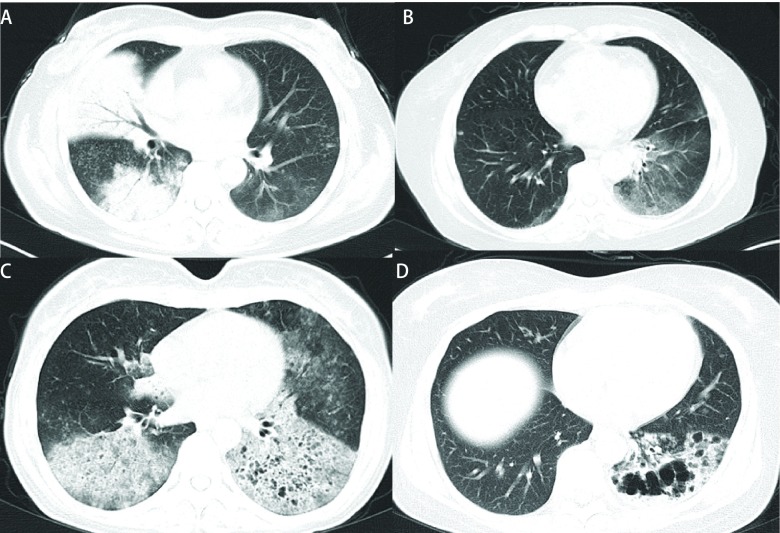
肺炎型肺癌的影像学表现。A：右中下肺多发实变影伴支气管充气征，左下肺片状磨玻璃影，双肺多发磨玻璃结节。B：双肺多发片状磨玻璃影，伴左侧叶间胸膜增厚, 右下肺小结节。C：双肺弥漫磨玻璃影伴多发囊性改变，双肺多发磨玻璃结节。D：左下肺磨玻璃实变影中有囊性破坏及空洞形成。 Imaging findings of pneumonic-type lung carcinoma. A: Consolidation with air-bronchogram in the right middle and lower lobes. Ground-glass attenuation in the left lower lobe. Multiple ground-glass nodules in both lungs. B: Ground-glass attenuation in both lunges with left interlobar pleural thickening. Note a small nodule in the right lower lobe. C: Diffuse ground-glass attenuation in both lungs, with multiple bubble-like low attenuation and ground-glass nodules. D: Consolidation and ground-glass attenuation in the left lower lobe with multiple bubble-like low attenuation and cavitation.

**1:** 肺炎型肺癌的肺部CT影像学表现 Chest CT features of pneumonic-type lung carcinoma

Chest CT features	Number of patients	Percentage
Ground glass attenuation	40	87.0%
Patchy consolidation	39	84.8%
Air-bronchogram	37	80.4%
Bronchial leafless tree sign	29	63.0%
Multiple ground-glass nodules	39	84.8%
Interlobular fissure edge bulging	15	32.6%
Bubble-like low attenuation	17	40.0%
Cavity	6	13.0%
Nodule or mass	9	19.6%
CT angiogram sign (34 cases)	22	64.7%
Different ipsilateral lobe pulmonary nodules	43	93.5%
Contralateral pulmonary nodules	39	84.8%
Pleural metastasis	16	34.8%
Mediastinal lymphadenopathy	29	63.0%
CT: computed tomography.		

### 2.4 病理诊断及分子分型

从发生症状到明确病理诊断的中位时间为214天（95%CI: 129天-298天）（图 2A）。16例患者CT引导下经皮肺穿刺，5例患者通过胸腔镜肺活检或手术获得病理，均明确诊断（确诊率100%）。33例患者完成支气管镜检查，23例明确诊断（确诊率69.7%）；其中21例行经支气管镜肺活检（transbronchial lung biopsy, TBLB），15例明确病理诊断（确诊率71.4%），31例支气管肺泡灌洗（bronchoalveolar lavage, BAL），16例获得病理诊断（确诊率51.6%）。能够同时完成TBLB和BALF的21例患者，17例明确诊断（确诊率80.9%）。20例患者痰液检查，其中9例痰中找到瘤细胞（确诊率45.0%）。

**2:**
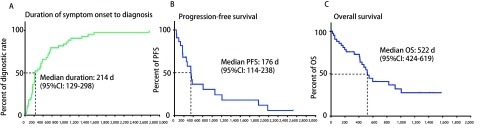
肺炎型肺癌患者自出现症状至确诊的时间（A），化疗相关无疾病进展时间（B）及总生存期（C）。 The duration from symptom onset to diagnosis (A), progression-free survival for patients underwent chemotherapy (B), and the overall survival of pneumonic-type lung carcinoma patients (C).

病理分型，26例（26/46, 56.5%）为浸润性肺粘液腺癌，另外20例（20/46, 43.5%）患者因为活检标本太小，或者为细胞学标本，仅诊断为腺癌，无法进一步区分病理亚型。5例外科肺活检的患者均明确分型为浸润性粘液腺癌。对38例进行了*EGFR*基因检测，仅6例（6/38, 15.8%）检测到*EGFR*突变。33例*ALK*基因检测，仅1例（1/33, 3%）有*ALK*基因重排。另有3例检测到*KRAS*突变，1例患者检测到*BRAF* V600E突变。在明确诊断粘液腺癌的26例患者中，22例完成*EGFR*基因检测，仅1例（1/22, 4.5%）有*EGFR*突变exon19 del；17例做*ALK*基因检测，仅1例（1/17, 5.9%）患者有ALK重排。

### 2.5 临床分期

41例患者诊断为Ⅳ期。仅5例诊断Ⅲ期，但是4例为同侧不同肺叶转移，1例为肿瘤巨大，上述5例患者无法行手术治疗及放疗。45例（45/46, 97.8%）患者有同侧和/或对侧肺转移。发生胸腔外转移者有5例，其中4例为骨转移，1例腹股沟淋巴结转移。

### 2.6 治疗及预后

由于此类患者大多数无明确驱动基因，因此化疗是主要治疗策略。29例患者接受化疗，其化疗方案22例为培美曲塞联合铂类，2例应用吉西他滨联合顺铂方案化疗，3例紫杉醇联合铂类方案化疗，1例应用长春瑞滨联合铂类方案化疗，1例化疗方案不详，有4例患者联合应用贝伐珠单抗治疗。疗效评价方面，3例患者疗效部分缓解（partial response, PR），17例患者疗效稳定（stable disease, SD），5例患者疾病进展（progressive disease, PD），4例疗效不详。患者的中位PFS为176天（95%CI: 114-238）（图 2B）。18例患者接受靶向治疗，其中6例*EGFR*突变患者，1例疗效评价PR，疗效评价为5例SD，5例患者PFS时间分别为5.7个月、15个月、20个月、31个月和44个月，1例患者失访；1例ALK阳性，先后应用塞瑞替尼及克唑替尼治疗，最佳疗效SD，总的疾病控制时间目前已达48个月；两例应用安罗替尼患者，最佳疗效SD，PFS时间分别为5个月及6个月；其他无明确驱动基因的患者，试用靶向治疗药物均失访或疾病进展。截止至2019年1月16日末次随访，24例患者死亡，18例存活，4例失访。本组患者自诊断晚期肺癌后，中位OS为522天（95%CI: 424-619）（图 2C）。接受化疗的患者中位OS 547天（95%CI: 446-648），较非化疗组中位OS 406天（95%CI: 280-532）略有延长（HR=0.676, *P*=0.387, 3）（图 3A）。有*EGER*突变或*ALK*融合基因的患者生存时间较长，该组患者中位生存期尚未达到，较EGFR野生型或*ALK*融合基因阴性患者中位OS 498天（95%CI: 404-591）略有延长（HR=0.444, *P*=0.106, 5）（图 3B）。而EGFR野生型或*ALK*融合基因阴性的患者，接受化疗患者的中位OS 547天（95%CI: 492-602），不接受化疗患者的中位OS 331天（95%CI: 22-919），化疗使中位OS明显延长（HR=0.155, *P*=0.002, 2）（图 3C）。药物治疗后，患者的痰液量明显减少，生活质量显著改善。

**3:**
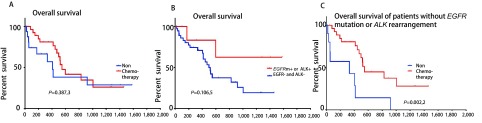
肺炎型肺癌患者的总生存期。A：化疗组患者对比无化疗组患者的*Kaplan-Meier*生存曲线；B：*EGFR*突变或ALK重排的患者对比无*EGFR*突变或ALK重排患者的*Kaplan-Meier*生存曲线；C：无EGFR或ALK重排的患者，化疗组对比无化疗组的*Kaplan-Meier*生存曲线。 Overall survival in patients with pneumonic-type lung carcinoma. A: *Kaplan-Meier* estimates in patients with chemotherapy versus non-chemotherapy. B: *Kaplan-Meier* estimates in patients with *EGFR* mutation or ALK rearrangement versus patients without *EGFR* mutation or ALK rearrangement. C: *Kaplan-Meier* estimates in patients without *EGFR* mutation or ALK arrangement with chemotherapy versus non-chemotherapy.

## 3 讨论

肺炎型肺癌是一种特殊临床表现的肺癌，通常到确诊时已是晚期，治疗上进展少。本研究对肺炎型肺癌的临床资料进行总结分析，以期能够更多的挖掘临床特征，指导临床工作。本研究的结果显示：（1）咳嗽、咯白泡沫痰为肺炎型肺癌的主要临床表现，肺部影像表现为磨玻璃影及实变影等肺泡填充病变，少有肿块。（2）支气管镜下的肺活检（TBLB）配合支气管肺泡灌洗（BAL）的诊断率相当高（80.9%），而经皮肺穿刺活检及外科肺活检均有100%的诊断率。（3）浸润性粘液腺癌是肺炎型肺癌的主要病理亚型，绝大部分患者有肺内转移。（4）肺炎型肺癌的*EGFR*突变率很低。（5）有驱动基因的患者生存期相对长，无驱动基因的患者，化疗可延长生存期。

此类患者痰液多以白色泡沫痰为主。部分患者病程中有间断发热，黄痰，可能为合并感染所致。抗感染后黄痰消失，但仍持续有咳白痰，且随着病情进展，咳痰量逐渐增多。因此，对于中老年患者，肺部浸润影经抗感染治疗不吸收，且持续咳嗽、咯大量白痰者，应警惕肺炎型肺癌之可能，并积极获取病理学诊断。

肺部CT可提供鉴别诊断线索。Kim等^[7]^曾比较肺部感染和类似于肺炎表现的细支气管肺泡癌的影像学表现，结果显示，囊泡样改变（bubble-like low-attenuation）对于恶性病变有提示意义，而病变附近的胸膜增厚和支气管管壁增厚则对于肺炎更有提示意义。由于肿瘤细胞对于肺结构的破坏或者细支气管-肺泡堵塞产生活瓣效应，导致在磨玻璃或者实变影中，出现囊样破坏或多发空洞样融合，此类影像学表现高度提示肺炎型肺癌。本研究40%的病例肺部CT可见多发囊样变，13%的病例有空洞。此外，由于肿瘤细胞的转移特征，可能出现同侧或对侧肺的转移性结节，尤其是多发磨玻璃结节。本研究中84.8%的病例均有磨玻璃结节。另外，由于肿瘤的增生和粘液的分泌，可以出现叶间裂膨胀或者枯树枝征，这在肺部感染中也较为少见。

对于怀疑肺炎型肺癌的患者，应积极活检明确诊断。支气管镜是比较简单易行的操作。虽然部分患者通过BALF可获得病理诊断，为了提高诊断率，明确病理分型以及进一步基因检测，应尽可能做TBLB。但由于TBLB为盲检小标本，因此仍有可能无法明确诊断。CT引导的肺穿刺活检对肺炎型肺癌诊断率很高，主要风险为气胸。本组患者中一例患者穿刺后出现气胸，需要进一步处理。另外，由于部分患者就诊时已经有呼吸衰竭，可能无条件进行有创操作，收集痰液找瘤细胞也可以作为一种策略，反复留取，诊断率亦可达45%。

肺炎型肺癌的患者病理以粘液腺癌为多见。但是，影像学的肺炎型肺癌是否与病理上的肺粘液腺癌完全划等号呢？Watanabe等^[8]^分析了肺粘液腺癌的临床影像学表现，结果显示75%患者（30/40）为孤立的结节或肿块，而25%（10/40）为肺炎型肺癌。Cha等^[4]^研究Ⅳ期肺粘液腺癌，同样可表现为肿块型、肿块联合实变型及肺炎型，其中晚期患者肺炎型占50%。上述研究显示，并非所有的肺粘液腺癌均表现为肺炎型肺癌。Cha等^[4]^的研究同时还显示，在浸润性非粘液腺癌，2.9%（6/210）的患者影像学也可表现为肺炎型。也就是说，肺炎型肺癌并非全部都是粘液腺癌。我们的研究中，活检标本量足够的病例均确诊为浸润性粘液腺癌，而其余病例因缺少大的活检标本，仅能诊断为腺癌。但是有个例的病例，镜下表现为混合型，部分有粘液成分。另外，本研究中也有两例患者，镜下见到了微乳头结构，但由于活检标本小，无法完全进行病理亚型的区分。综上，我们认为肺炎型肺癌以粘液腺癌多见，但是，肺炎型肺癌并不完全等同于粘液肺腺癌。另外，此类肺癌血管侵袭少见（1/10），而气腔内播散较多见（7/10）^[8]^，这或许可以解释此类肺癌多有肺内转移^[4]^，而少有肺外转移。

有基因突变的肺腺癌患者采用相应的靶向治疗可延长生存期，这是公认的治疗策略。但是，肺炎型肺癌/粘液腺癌常见的肺癌驱动基因突变的发生率极低。本研究中明确分型为粘液腺癌的患者，*EGFR*突变发生率仅为4.5%，*ALK*突变仅为5.9%。然而中国的肺腺癌患者*EGFR*总体突变率在40%-50%，故此类肺炎型肺癌/肺粘液腺癌可能具有独特的基因表型。有多项研究总结了肺粘液腺癌的基因检测特点^[8-10]^。*KRAS*是最常见的突变类型，约占35%-75%^[4, 8-10]^，*TP53*的突变发生率约46%^[10]^，而*EGFR*突变、ALK重排、*BRAF*突变的发生率非常低。浸润性肺粘液腺癌*KRAS*突变发生率尽管较高，且KRAS与细胞外粘蛋白有相关性，但是并无明确的靶向治疗策略^[11]^。2014年，Fernandez-Cuesta等^[12]^研究发现一种新的融合基因，*CD74-NRG1*融合，在102例无常见基因突变的肺癌中，检测到5例*CD74-NRG1*基因，而且这5例患者均为浸润性粘液腺癌。研究证实这种融合基因也是一种明确的驱动基因，并且可以针对性的制定靶向治疗的药物。Nakaoku等^[13]^研究了90例浸润性粘液腺癌的基因表型，56例有*KRAS*基因突变。在另外34例无*KRAS*突变粘液腺癌肿瘤中，*NRG1*重排的发生率为17.6%（6/34），另有2例*BRAF*突变，*EGFR*突变、ALK重排、RET重排、BRAF重排及ERBB4重排各有1例。Shin^[14]^研究了59例浸润性粘液腺癌患者的基因表型，共检测到16例*NRG1*融合基因（13例*SLC3A2-NRG1*和3例*CD74-NRG1*），其中10例同时有*KRAS*突变，而且有*NRG1*融合基因的患者预后更差。后续的研究也在粘液腺癌患者中检测到*CD74-NRG1*融合基因，但是都是相对较少的案例报道^[15, 16]^。Guo等^[17]^分析了粘液腺癌的免疫检查点特征基因是VTCN1/B7-H4而非PD-L1/B7-H1，在有*KRAS*基因突变的情况下，转录因子FOXA3和SPDEF可诱导肺粘液腺癌。综上，对于肺炎型肺癌/粘液腺癌，*KRAS*突变发生率高，*EGFR*突变和ALK重排发生率低，*CD74-NRG1*融合基因可能是潜在的驱动基因，需要更进一步的基因表型方面的研究和探索，并制定相应的靶向治疗药物。

治疗上，本研究中化疗对肺炎型肺癌的疾病控制率可达80%，经过化疗的患者生存期显著延长，而且可观察到用药后痰量的减少。因此，虽然此类患者多属于晚期，仍建议积极的治疗。尽管有文献报道含铂方案对此类患者的疗效不尽如人意，但是培美曲塞的疗效值得关注。本研究中一线治疗患者的化疗方案以培美曲塞联合铂类为主，因此获得了比较满意的生存时间延长^[4]^。值得关注的是，单药安罗替尼的患者，也可以达到5个月-6个月的PFS。我们临床上还观察到两例应用贝伐珠单抗联合化疗的患者，用药后痰液量明显减少，提示或许抗血管生成治疗对此类患者的症状及生活质量改善有帮助。此外，对于有明确*EGFR*或*ALK*驱动基因的患者，靶向治疗仍能够延长患者生存期。遗憾的是此类患者突变率很低，无基因突变的患者靶向治疗效果差^[4]^。

肺炎型肺癌主要病理类型是肺浸润性粘液腺癌。对于有肺部阴影，临床初诊为肺炎而抗感染无效的患者，若肺部CT在片状磨玻璃影-实变的基础上同时有多发囊泡、空洞或磨玻璃结节，则需要高度怀疑肺炎型肺癌，应该积极取活检确诊，并明确进一步病理分型及分子分型。对于有明确驱动基因的患者，可以积极的给予靶向治疗。无驱动基因的患者，化疗仍可延长患者生存，并明显减少咯痰症状。抗血管生成治疗或许对缓解咳痰症状有效。

## References

[1] Siegel RL, Miller KD, Jemal A (2017). Cancer Statistics, 2017. CA Cancer J Clin.

[2] Li T, Kung HJ, Mack PC (2013). Genotyping and genomic profiling of non-small-cell lung cancer: implications for current and future therapies. J Clin Oncol.

[3] Travis WD, Brambilla E, Noguchi M (2011). International association for the study of lung cancer/american thoracic society/european respiratory society international multidisciplinary classification of lung adenocarcinoma. J Thorac Oncol.

[4] Cha YJ, Kim HR, Lee HJ (2016). Clinical course of stage Ⅳ invasive mucinous adenocarcinoma of the lung. Lung Cancer.

[5] Detterbeck FC, Boffa DJ, Tanoue LT (2009). The new lung cancer staging system. Chest.

[6] Eisenhauer EA, Therasse P, Bogaerts J (2009). New response evaluation criteria in solid tumours: revised RECIST guideline (version 1.1). Eur J Cancer.

[7] Kim TH, Kim SJ, Ryu YH (2006). Differential CT features of infectious pneumonia versus bronchioloalveolar carcinoma (BAC) mimicking pneumonia. Eur Radiol.

[8] Watanabe H, Saito H, Yokose T (2015). Relation between thin-section computed tomography and clinical findings of mucinous adenocarcinoma. Ann Thorac Surg.

[9] Boland JM, Maleszewski JJ, Wampfler JA (2018). Pulmonary invasive mucinous adenocarcinoma and mixed invasive mucinous/nonmucinous adenocarcinoma-a clinicopathological and molecular genetic study with survival analysis. Hum Pathol.

[10] Righi L, Vatrano S, Di Nicolantonio F (2016). Retrospective multicenter study investigating the role of targeted next-generation sequencing of selected cancer genes in mucinous adenocarcinoma of the lung. J Thorac Oncol.

[11] Kadota K, Yeh YC, D'Angelo SP (2014). Associations between mutations and histologic patterns of mucin in lung adenocarcinoma: invasive mucinous pattern and extracellular mucin are associated with *KRAS* mutation. Am J Surg Pathol.

[12] Fernandez-Cuesta L, Plenker D, Osada H (2014). CD74-NRG1 fusions in lung adenocarcinoma. Cancer Discov.

[13] Nakaoku T, Tsuta K, Ichikawa H (2014). Druggable oncogene fusions in invasive mucinous lung adenocarcinoma. Clin Cancer Res.

[14] Shin DH, Lee D, Hong DW (2016). Oncogenic function and clinical implications of SLC3A2-NRG1 fusion in invasive mucinous adenocarcinoma of the lung. Oncotarget.

[15] Duruisseaux M, McLeer-Florin A, Antoine M (2016). NRG1 fusion in a French cohort of invasive mucinous lung adenocarcinoma. Cancer Med.

[16] Duruisseaux M, Antoine M, Rabbe N (2017). Lepidic predominant adenocarcinoma and invasive mucinous adenocarcinoma of the lung exhibit specific mucin expression in relation with oncogenic drivers. Lung Cancer.

[17] Guo M, Tomoshige K, Meister M (2017). Gene signature driving invasive mucinous adenocarcinoma of the lung. EMBO Mol Med.

